# Chebulinic acid inhibits smooth muscle cell migration by suppressing PDGF-Rβ phosphorylation and inhibiting matrix metalloproteinase-2 expression

**DOI:** 10.1038/s41598-017-12221-w

**Published:** 2017-09-18

**Authors:** In-Sung Song, Yu Jeong Jeong, Jung-Hyun Park, Sungbo Shim, Sung-Wuk Jang

**Affiliations:** 1 Department of Biomedical Sciences, University of Ulsan College of Medicine, Seoul, 138-736 Korea; 2 Department of Biochemistry and Molecular Biology, University of Ulsan College of Medicine, Seoul, 138-736 Korea; 3 Department of Biochemistry, Neuromarker Resource Bank, College of Natural Sciences, Chungbuk National University, Cheongju, Republic of Korea

## Abstract

Excessive migration of vascular smooth muscle cells (VSMCs) after vascular injury contributes to the development of occlusive vascular disease. Inhibition of VSMC migration is a validated therapeutic modality for occlusive vascular diseases, such as atherosclerosis and restenosis. We investigated the inhibitory effect of chebulinic acid (CBA) on cell migration and matrix metalloproteinase (MMP)-2 activation in platelet-derived growth factor (PDGF)-BB-induced mouse and human VSMCs. CBA significantly inhibited PDGF-BB-induced migration in mouse and human VSMCs, without inducing cell death. Additionally, CBA significantly blocked PDGF-BB-induced phosphorylation of the PDGF receptor (PDGF-R), Akt, and extracellular signal-regulated kinase (ERK)1/2 by inhibiting the activation of the PDGF-BB signalling pathway. In both mouse and human VSMCs, CBA inhibited PDGF-induced MMP-2 mRNA and protein expression as well as the proteolytic activity of MMP-2. Moreover, CBA suppressed sprout outgrowth formation of VSMCs from endothelium-removed aortic rings as well as neointima formation following rat carotid balloon injury. Taken together, our findings indicated that CBA inhibits VSMC migration by decreasing MMP-2 expression through PDGF-R and the ERK1/2 and Akt pathways. Our data may improve the understanding of the antiatherogenic effects of CBA in VSMCs.

## Introduction

Atherosclerosis is a blood vessel disorder in which arteries thicken because of the accumulation of cholesterol, lipids, vascular smooth muscle cells (VSMCs) and immune cells^1–3^. VSMC proliferation and migration contribute to the progression of atherosclerosis, restenosis after angioplasty and stent placement, and vein graft failure^4,5^. The migratory and proliferative activities of VSMCs are regulated by multiple factors, such as growth factors, cytokines, and matrix metalloproteinases (MMPs) in the microenvironment of atherosclerotic lesions^6,7^.

Among the various growth factors, platelet-derived growth factor (PDGF), which is released by platelets, endothelial cells, and many other cells at the site of injury, is the most potent VSMC mitogen^8^. The role of PDGF in the pathogenesis of arterial injury disorders, including atherosclerosis and post-angioplasty restenosis, has been well established^8–10^. The binding of PDGF to the PDGF receptor (PDGF-R) activates various downstream signalling proteins, including those involved in the phospholipase C (PLC)-γ1, extracellular signal-regulated kinase (ERK)1/2, and phosphatidylinositol 3 kinase (PI3K)/Akt pathways^11^. Mitogen-activated protein kinase (MAPK) signalling also plays an important role in the regulation of proliferation, migration and survival of mammalian cells^12^. Akt is a downstream target of PI3K and plays a pivotal role in cell migration, growth and anti-apoptotic events in various cell types^13,14^. Akt has been shown to enhance MMP expression and activity *in vitro* ^15,16^. Consequently, the upregulation of PDGF signalling can instigate the development and progression of cardiovascular diseases, such as hypertension and atherosclerosis^10^. Therefore, modulating the PDGF downstream signalling pathway in VSMCs may be a favourable strategy for the prevention of atherosclerosis.

For VSMC migration to occur, proteolytic degradation or remodelling of the extracellular matrix (ECM) is required. MMPs catalyse and remove the basement membrane around VSMCs and facilitate their contact with the interstitial matrix^17,18^. MMPs are a family of zinc-dependent endopeptidases that regulate a wide variety of processes associated with vascular structure and remodelling^7^. In particular, MMP-2 plays a critical role in VSMC migration and neointima formation, and PDGF is known to activate VSMC migration by inducing MMP-2 expression^19^. In a mouse model of restenosis, MMP-2-deficient mice exhibited reduced neointima formation due to a defect in VSMC migration^20^, suggesting that MMP-2 plays a critical role in the fate of atherosclerosis.

**Figure 1:**
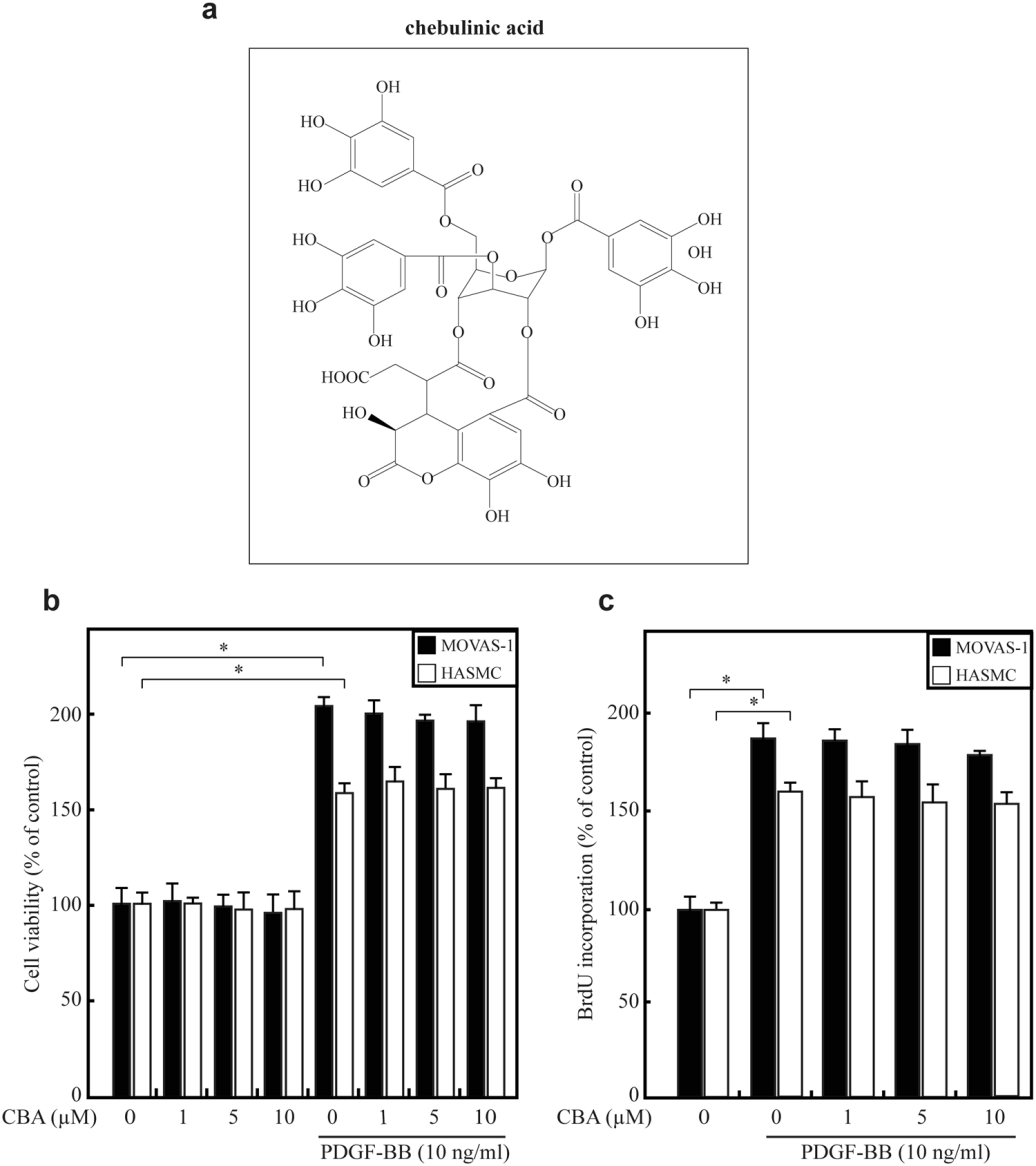
Effect of CBA on the proliferation of HASMCs and MOVAS-1 cells. (**a**) Chemical structure of CBA. (**b**) HASMCs (white bars) and MOVAS-1 cells (black bars) pretreated with pretreated with 0–10 μΜ CBA with or without 10 ng/ml PDGF-BB for 24 h. Cell viability was measured using an MTT assay; *P < 0.05 versus vehicle-treated cells. Data represent the mean ± SE of three independent experiments. (**c**) Serum-starved HASMCs (white bars) and MOVAS-1 cells (black bars) were stimulated with PDGF-BB in the presence of the indicated concentrations of CBA for 24 h, and subsequently, the incorporation of BrdU was determined; *P < 0.05 versus vehicle-treated cells. Data represent the mean ± SE of three independent experiments.

Recent studies have emphasised the pathogenic role and potential therapeutic implications of natural compounds in atherosclerosis^21^. Chebulinic acid (CBA; Fig. 1a) is a natural hydrolysable tannin widely present in several medicinal plants, such as *Phyllanthus emblica*, *Terminalia arborea* and *T. chebula* ^22,23^. CBA is a pleiotropic compound showing anti-tumour^24^, anti-angiogenic^25^, anti-inflammatory^26^ and anti-hypertensive^27^ effects in numerous cellular and *in vivo* models. Although it is most commonly used as an antioxidant agent, it was recently reported to be effective in VEGF-mediated angiogenesis and transforming growth factor (TGF)-β-induced epithelial-mesenchymal transition (EMT)^25,28^. Although there have been reports of the inhibitory effects of CBA on endothelial and epithelial cell migration as well as angiogenesis and EMT, no study has reported its effect on the cellular response to PDGF, which is the main growth factor regulating post-angioplasty VSMC migration. However, the effect of CBA on smooth muscle cell (SMC) migration has not yet been clarified. In the present study, we investigated the inhibitory effect of CBA on PDGF-BB-induced VSMC migration and the potential mechanisms involved using *in vitro* and *in vivo* experiments.

## Results

### CBA suppresses the migration of VSMCs

Because PDGF-induced proliferation of VSMCs is critical in atherosclerotic lesion formation and post-angioplasty restenosis^10^, we first determined the effect of CBA on PDGF-BB-induced proliferation of mouse aortic SMCs (MOVAS-1 cells) and human aortic SMCs (HASMCs). PDGF treatment for 24 h significantly increased the viability of both MOVAS-1 cells and HASMCs. As shown in Fig. 1b, CBA pretreatment (1–10 μM) did not affect cell viability, assessed using an MTT assay, indicating that CBA is safe for both MOVAS-1 cells and HASMCs. When the amount of the newly synthesised DNA was quantified by measuring incorporated 5-bromo-2′-deoxyuridine (BrdU) in HASMCs and MOVAS-1 cells treated with PDGF and CBA for 24 h, CBA did not affect PDGF-induced DNA synthesis (Fig. 1c). Therefore, this concentration range was used in all subsequent experiments.

**Figure 2:**
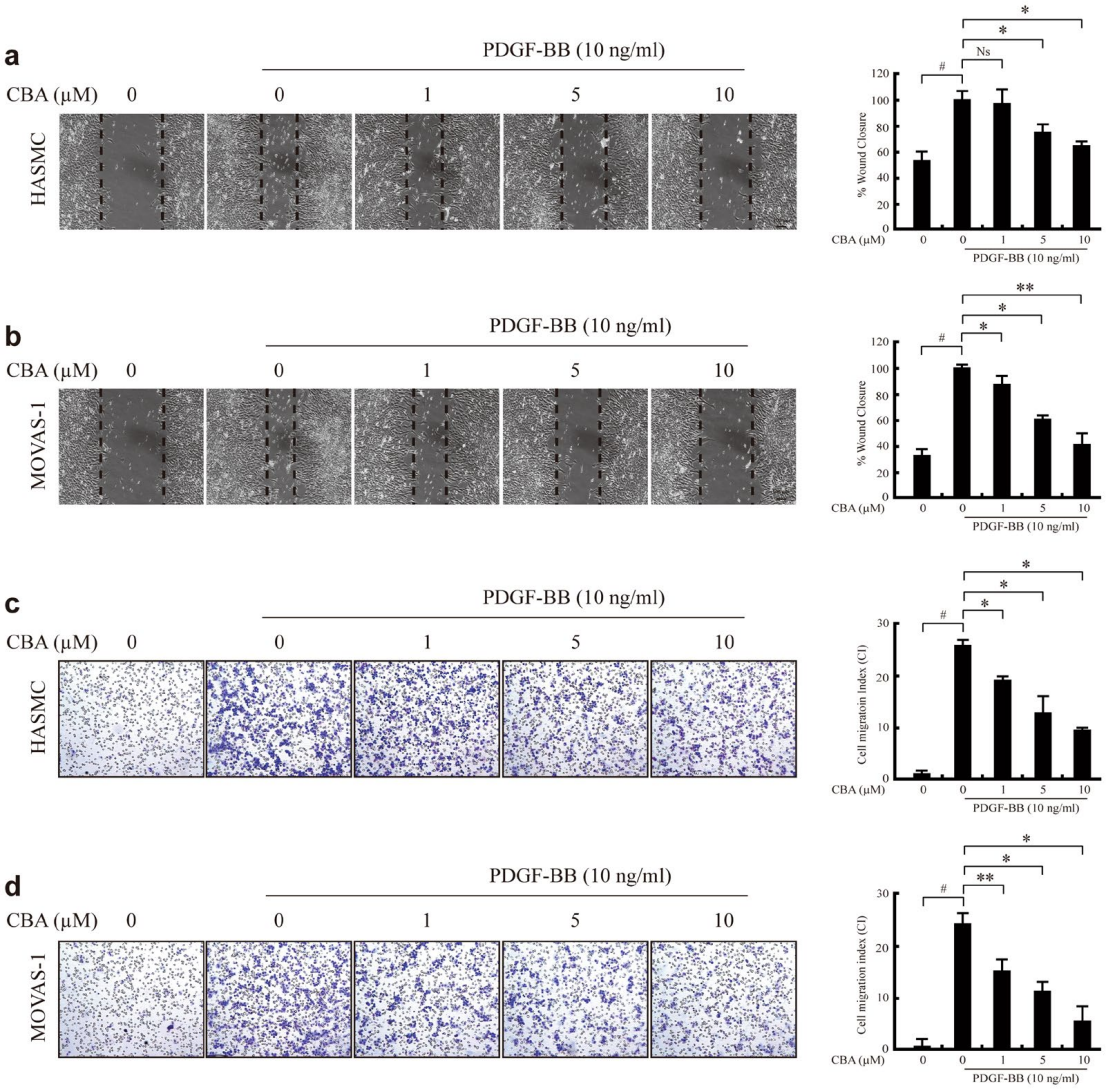
CBA inhibits PDGF-BB-induced migration of HASMCs and MOVAS-1 cells. HASMCs (**a**) and MOVAS-1 cells (**b**) were scratched with a pipette tip and incubated with various concentrations of CBA in serum-free medium. After 30 min, the cells were treated with 10 ng/ml PDGF-BB for 24 h (MOVAS-1 cells) or 48 h (HASMCs). Representative images of wound healing were taken at the time of scratching and 24 h (or 48 h) after wounding. Original magnification, 100 × . Scale bar, 100 μm. Wound healing was quantified as the percentage of cells migrating into the wound with respect to the total number of cells; ^*#*^ *P* < 0.001 versus vehicle-treated cells, *P < 0.01 versus PDGF-treated cells, Ns: no significant. Data represent the mean ± SE of three independent experiments. HASMCs (**c**) and MOVAS-1 (**d**) were pre-incubated with CBA at the indicated concentrations for 4 h and treated with or without PDGF in a microchemotaxis chamber. After incubation for 2 h, filters were stained with Diff-Quick. Migrated cells were counted in six randomly selected fields per well. The chemotactic index (CI) was calculated from the number of cells that migrated compared to vehicle control. Results are expressed as the mean CI ± SEM of six replicate measurements from a single experiment and are representative of three independent experiments; ^*#*^ *P* < 0.001 versus vehicle-treated cells, *P < 0.01 versus PDGF-treated cells.

In atherosclerosis, the balance between migration and proliferation of VSMCs is an important factor in plaque stability^29^. To investigate the effect of CBA on VSMC migration, we conducted a wound-healing assay using both HASMC and MOVAS-1 cells. After a scratch wound was made in a cell monolayer, the effects of various concentrations of CBA on PDGF-BB-induced cell migration were examined. The cells were allowed to migrate for 24 h (MOVAS-1) or 48 h (HASMCs), after which the migration distances were measured. As shown in Fig. 2a and b, CBA suppressed the PDGF-BB-induced migration of both HASMCs (Fig. 2a) and MOVAS-1 cells (Fig. 2b). We also determined that CBA inhibited VSMC migration when added after the challenge with PDGF-BB. The results showed no significant difference before and after PDGF treatment (Supplemental Figure S1).

To confirm these findings, we examined chemotaxis using a microchemotaxis chamber. CBA decreased both PDGF-induced human and mouse aortic VSMC migration in a dose-dependent manner (Fig. 2c and d).

### CBA prevents VSMC migration by inhibiting MMP-2 expression

**Figure 3:**
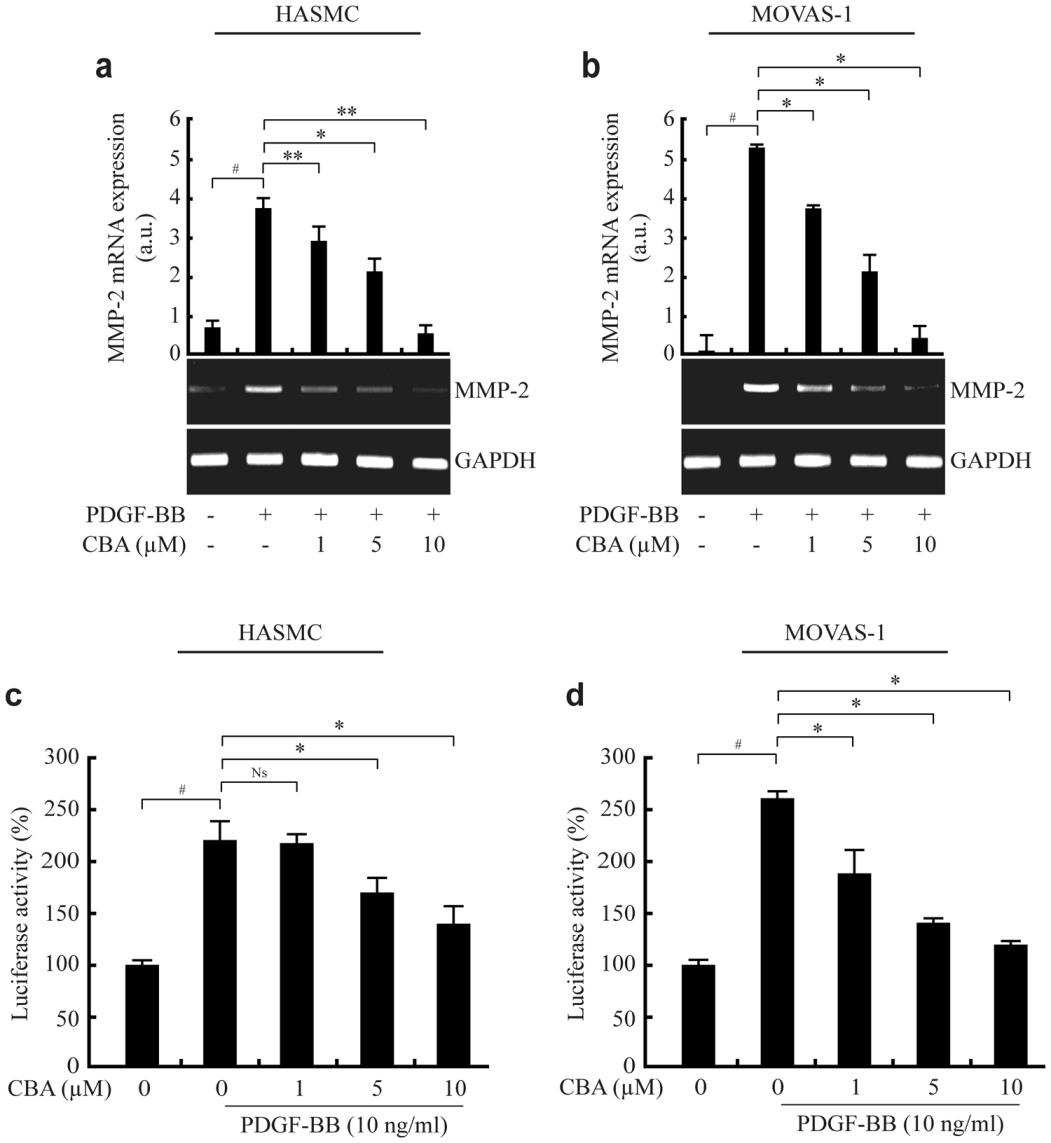
CBA reduces PDGF-induced MMP-2 gene expression in HASMCs and MOVAS-1 cells. HASMCs (**a**) and MOVAS-1 cells (**b**) were incubated with the indicated concentrations of CBA for 30 min followed by stimulation with 10 ng/ml PDGF-BB for 24 h. The mRNA level of endogenous MMP-2 was measured by RT-PCR. GAPDH was used as an internal control; ^*#*^ *P* < 0.001 versus vehicle-treated cells, *P < 0.01 versus PDGF-treated cells. Data represent the mean ± SE of three independent experiments. HASMCs (**c**) and MOVAS-1 cells (**d**) were transfected with pMMP-2-luciferase and pSV40-β-galactosidase vectors. The transfected cells were treated with the indicated concentrations of CBA for 30 min and stimulated with 10 ng/ml PDGF-BB for 9 h. Luciferase activity was normalised to β-galactosidase activity. Each value represents the mean ± SE of three independent experiments and is expressed relative to control; ^*#*^ *P* < 0.001 versus vehicle-treated cells, *P < 0.01 versus PDGF-treated cells, Ns: no significant.

**Figure 4:**
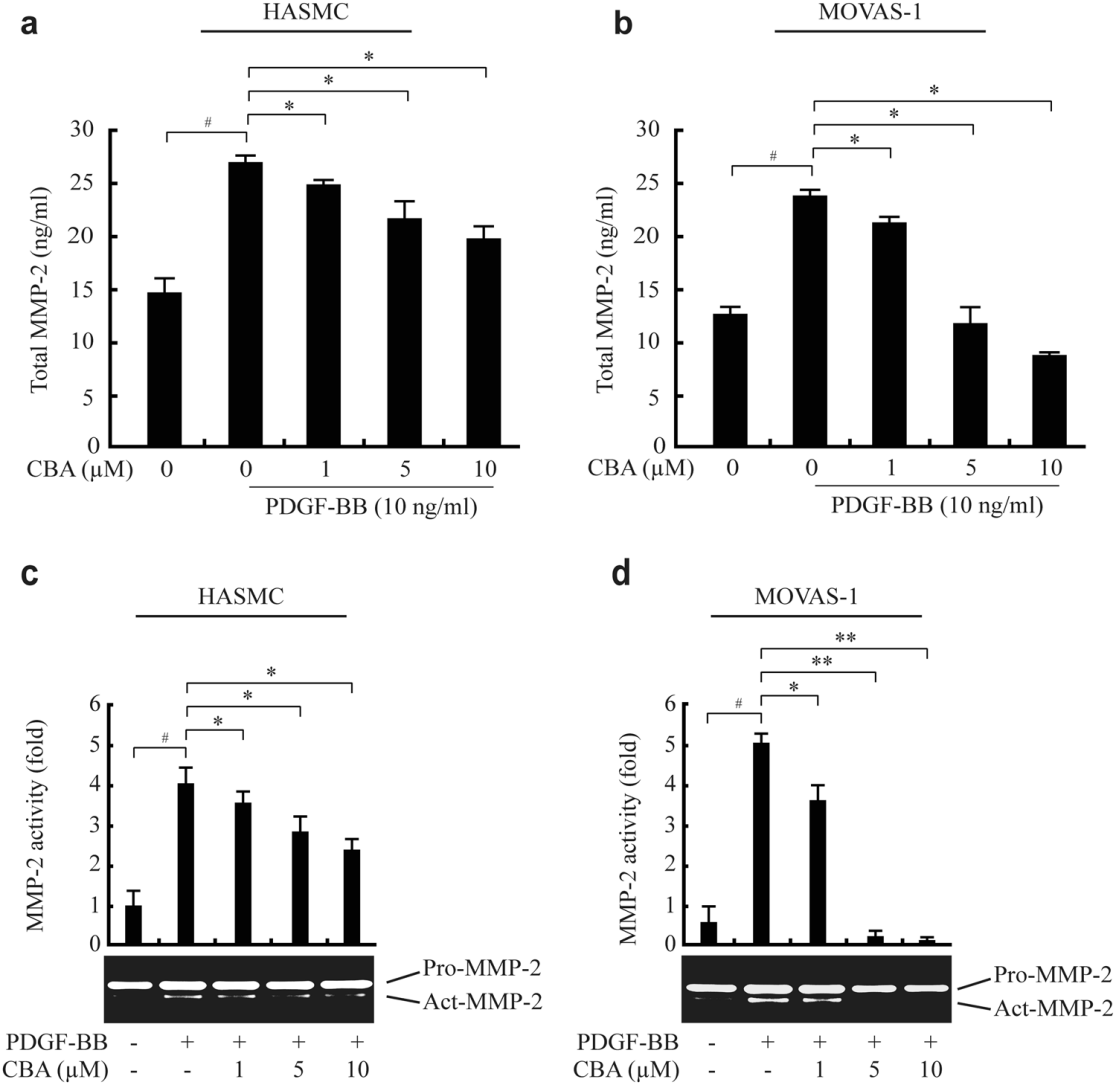
CBA inhibits PDGF-induced activity and secretion of MMP-2 in HASMCs and MOVAS-1 cells. HASMCs and MOVAS-1 cells were pre-treated with CBA for 30 min and stimulated with 10 ng/ml PDGF-BB for 24 h. The conditioned medium was collected and assayed for secreted MMP-2 using ELISA (**a** and **b**) and gelatin zymography (**c** and **d**); ^*#*^ *P* < 0.001 versus vehicle-treated cells, *P < 0.01 versus PDGF-treated cells. Data represent the mean ± SE of three independent experiments.

MMPs, which are involved in the degradation of ECM, are associated with the atherosclerosis process, from the initial lesion to plaque rupture^30^. In particular, gelatinase MMP-2-dependent vascular ECM degradation promotes VSMC migration and early plaque development^31^. The expression of MMPs is regulated by various inflammatory cytokines and growth factors^19^. Because PDGF is a potential inducer of MMP-2 and promotes cell migration, we examined the effect of CBA on MMP-2 expression in VSMCs. PDGF increased MMP-2 expression in both MOVAS-1 cells and HASMCs by approximately 3.5-fold and 5.2-fold, respectively, whereas CBA suppressed PDGF-induced MMP-2 expression in a dose-dependent manner (Fig. 3a and b). Results of a luciferase assay showed that CBA suppresses MMP-2 promoter activity, indicating that CBA reduced MMP-2 expression at the transcriptional level (Fig. 3c and d). We next examined the effects of CBA on the secretion and proteolytic activity of MMP-2 in conditioned media. Results from an enzyme-linked immunosorbent assay (ELISA) (Fig. 4a and b) and a gelatin zymography assay (Fig. 4c and d) showed that PDGF increased the secretion and enzymatic activity of MMP-2, respectively, whereas CBA dose-dependently suppressed the secretion and proteolytic activity of MMP-2 in both MOVAS-1 cells and HASMCs. Collectively, these findings indicated that CBA suppresses PDGF-BB-induced MMP-2 expression at both the protein and mRNA levels, thus inhibiting the enzymatic activity of MMP-2.

### CBA suppresses PDGF-R-ERK1/2 and the Akt signalling pathway

**Figure 5:**
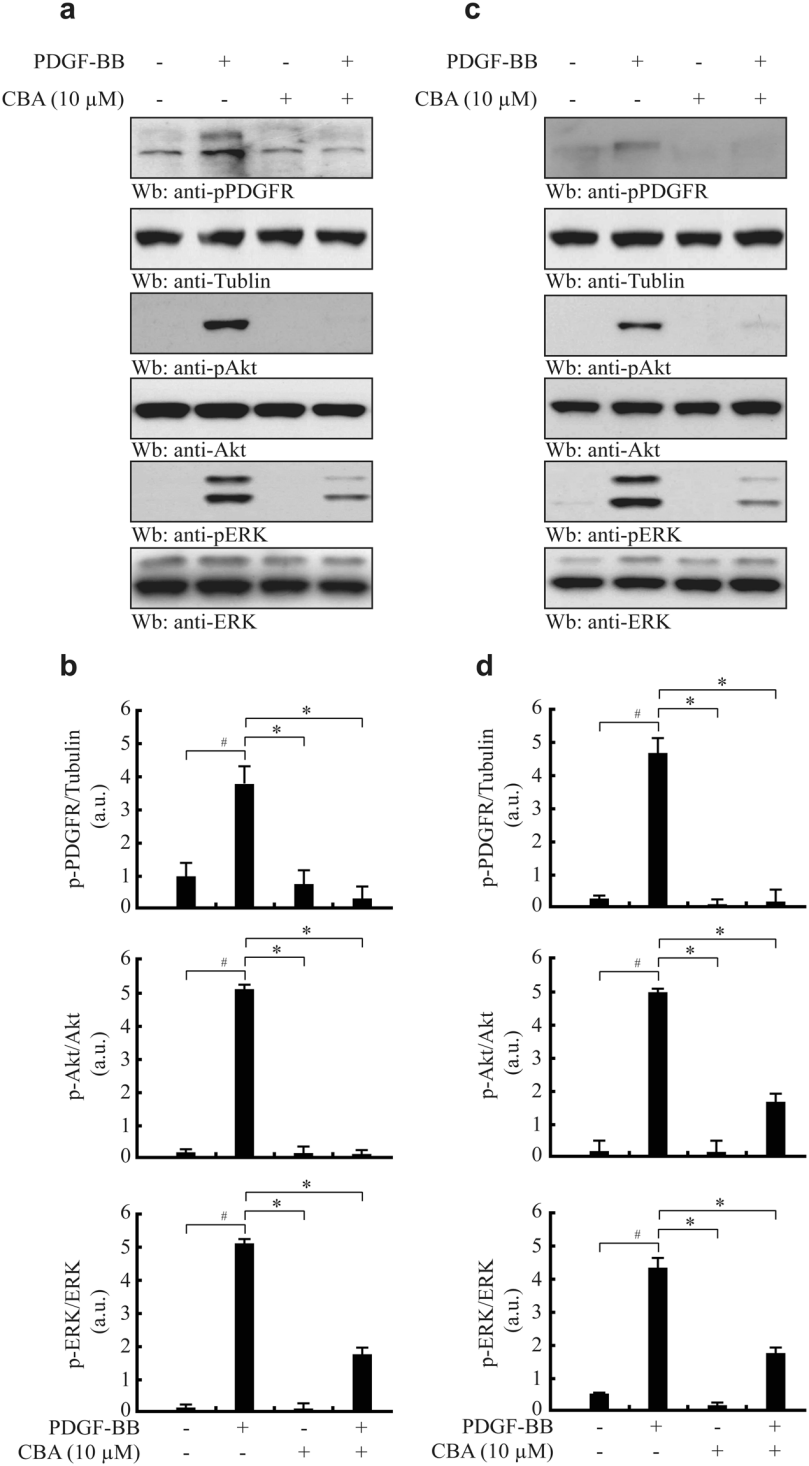
Effect of CBA on PDGF-induced activation of downstream signalling pathways. HASMCs (**a** and **b**) and MOVAS-1 cells (**c** and **d**) were pre-treated with CBA for 30 min and then stimulated with PDGF-BB for 15 min. Cell lysates were separated by 10% SDS-PAGE and subjected to western blotting with the indicated antibodies. The membrane was stripped and reprobed with anti-tubulin, anti-Akt, and anti-ERK as loading controls. Relative protein levels were quantified by densitometric scanning and normalised to the corresponding total protein from three independent experiments; ^*#*^ *P* < 0.001 versus vehicle-treated cells, *P < 0.01 versus PDGF-treated cells. Data represent the mean ± SE of three independent experiments.

PDGF, a potent mitogen, chemoattractant, and survival factor for VSMCs, is secreted by activated macrophages in atherosclerotic lesions. Activation of PDGF triggers the PI3K/Akt and Ras/MAPK signalling cascades^32^. We examined whether CBA selectively acts on the PDGF signalling pathway. To this end, we treated HASMCs (Fig. 5a and b) and MOVAS-1 cells (Fig. 5c and d) with 10 μM CBA followed by treatment with PDGF-BB. PDGF-BB markedly induced the phosphorylation of PDGF-R, which was substantially attenuated by treatment with CBA. In addition, CBA inhibited PDGF-induced ERK1/2 and Akt phosphorylation in both HASMCs and MOVAS-1 cells. These results indicated that CBA regulates VSMC migration through the inhibition of PDGF-R activation.

### CBA attenuates aortic ring outgrowth *ex vivo* and neointima formation following carotid balloon injury

**Figure 6:**
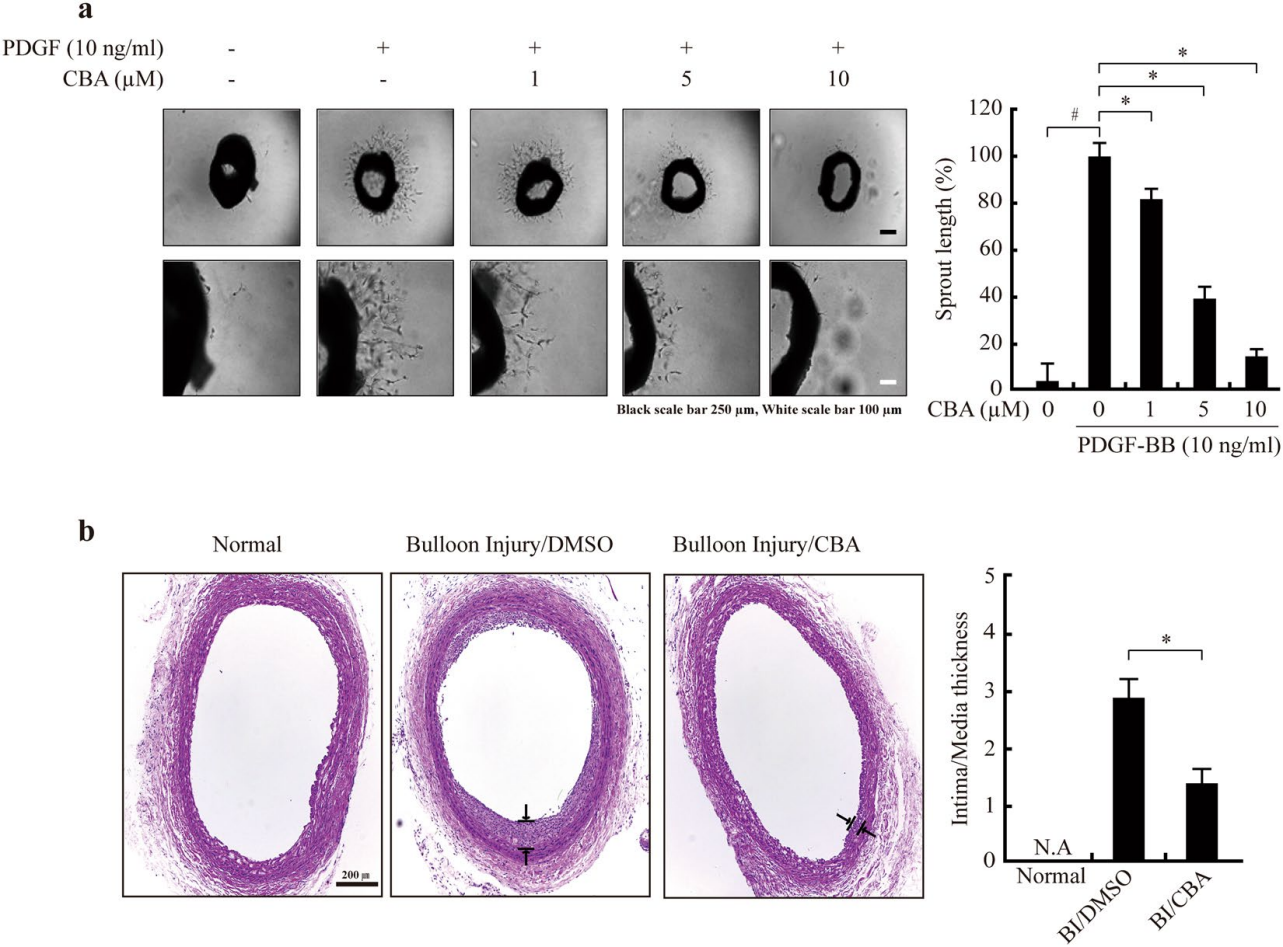
CBA attenuates VSMC migration *ex vivo* and *in vivo*. Sprout formation was determined by an aortic ring assay *ex vivo*. (**a**) Aortic rings were embedded in Matrigel in 48-well plates and treated with CBA and PDGF-BB in the culture medium for 3 days. Light microscopic images show the effects of CBA on PDGF-BB induced sprout formation. Quantitative analysis of the aortic ring assay is shown relative to the controls, and the data represent the mean ± SE of three experiments, ^*#*^ *P* < 0.001 versus vehicle-treated cells, *P < 0.01 versus PDGF-treated cells. (**b**) The effect of CBA on neointima formation following balloon injury (BI). The intima to media thickness ratio (I/M) was used to assess the effect of CBA on neointima formation. Arrows indicate neointima thickness. Quantification of the intimal area and I/M ratios of carotid arteries of rat from either the control group (DMSO) or the CBA-treated group is shwon [n = 6, *P < 0.01 versus injured control (DMSO)].

To examine the effect of CBA on VSMC migration in an animal model of atherosclerosis, we conducted an *ex vivo* aortic ring assay and assessed neointima formation after balloon injury (BI) *in vivo*. Aortic rings isolated from C57Bl/6 mice were embedded in Matrigel and the lengths of outgrowing sprouts were measured after 3 days. CBA at concentrations of 1, 5, and 10 μM reduced the sprout length by 20%, 62% and 83.8%, respectively (Fig. 6a). In addition, the effect of CBA on neointima formation was examined following rat carotid artery BI. Two weeks after BI, the intima to media thickness ratio (I/M ratio) significantly increased in the BI group, although such a BI-induced increase of the I/M ratio was significantly suppressed in the CBA-treated group (Fig. 6b). These results suggested that CBA markedly attenuates VSMC outhgrowth in *ex vivo* and *in vivo*.

## Discussion

To the best of our knowledge, this study was the first to provide evidence that: (i) CBA inhibits PDGF-induced VSMC migration *ex vivo* and *in vivo*, (ii) CBA inhibits the activation of PDGF-R downstream pathways, (iii) CBA regulates MMP-2 expression and activity and consequently, (iv) CBA inhibits PDGF-induced migration through the suppression of PDGF-R-mediated MMP-2 expression and proteolytic activity in VSMCs. These results indicate that CBA has a potent inhibitory effect on PDGF-BB-stimulated VSMC migration and may be useful for the treatment of vascular diseases and restenosis after angioplasty.

Several phenolic compounds with –OH groups tend to oxidise/breakdown in cell culture media and generate hydrogen peroxide (H_2_O_2_) or other oxidation products^33–35^. These products can exert effects on cells that have sometimes been mistakenly attributed to direct action of the treated compounds. Interestingly, CBA contains many –OH group, as depicted in Fig. 1a. To test the possibility that the effects of CBA were indirect, CBA, EGCG, resveratrol and curcumin were tested in an H_2_O_2_ generation assay. However, we could not detect significant H_2_O_2_ formation from CBA, which means that the cellular effects of CBA are unlikely to involve H_2_O_2_ production in the culture medium (Supplementary Figure S2). Presumably, CBA is stable in culture media. In addition, CBA doses used in this study (1–10 μM) appear to be in the appropriate range to exert its biological actions and are of physiological relevance given the plasma concentrations 2 μM CBA *in vivo* ^25^.

In mature vascular tissue, most VSMCs are in a quiescent and differentiated state within the medial layer of arteries. However, they can migrate into the intimal layer and proliferate after stimulation by growth factors and cytokines during atherosclerosis development and upon vascular injury^5,29^. Migration of VSMCs is a major cause of extensive intimal thickening and vessel wall inflammation^2^. Therefore, a number of studies have sought to identify the pathophysiological mechanism of VSMC migration and proliferation.

PDGF acts as a potent inducer of VSMC migration by phosphorylation of the PDGF receptor, which induces downstream signalling, including Akt and ERK1/2^11,36,37^. In this study, we showed that PDGF-BB-induced PDGF-R phosphorylation and downstream signalling were inhibited by CBA in both HASMCs and MOVAS-1 cells, suggesting that PDGF-R may be the direct target of CBA for inhibition of VSMC migration.

The induction of cell migration is regulated by early signals including Akt and ERK1/2, which can be promoted by PDGF-BB in VSMCs^32,37^. Akt contributes to ECM destruction by increasing the production of MMP-2 in VSMCs^38^. MMP-2 expression is associated with the induction of SMC hyperplasia during atherosclerosis and restenosis. Furthermore, MMP-2 plays a role in the migration of SMCs, which contributes to the intimal thickening of vascular lesions *in vivo* ^30,39^. Our results revealed that pretreatment with CBA inhibited the PDGF-induced increase in MMP-2 expression and its proteolytic activity in a dose-dependent manner.

Presumably, the suppressive effects of CBA on PDGF-induced Akt activation were associated with the subsequent decrease in MMP-2 mRNA expression and secretion. Based on these results, we suggest that the Akt pathway contributes to vascular cell migration. Our discovery is supported by a recent report that CBA inhibits EMT and MMP-2 induction in TGF-β-treated ARPE-19 cells. Conceivably, CBA might regulate Akt phosphorylation during TGF-β signalling.

It is well known that MAPK isoforms are common signals contributing to a variety of cell functions. PDGF increases MAPK activation and induces migration in several cell types, implying that MAPK signalling may be a crucial pathway in PDGF-mediated migration. Here, we show that CBA also inhibits PDGF-BB-induced phosphorylation of ERK1/2 in HVSMCs and MOVAS-1 cells, resulting in the inhibition of VSMC migration. These results imply that ERK1/2 as well as Akt are involved in the migration induced by PDGF-BB.

Phenotypic switching is an important phenomenon in VSMC activation. In response to vascular injury, the phenotype of VSMCs changes from quiescent, differentiated, and contractile to less differentiated and synthetic^40^. In addition to increased proliferation and migration, dedifferentiated VSMCs demonstrate decreased expression of smooth muscle-specific contractile markers, such as smooth muscle α-actin (SMC α-actin)^41^. It is well established that PDGF-BB is a key mediator of VSMC phenotypic switching^42^. In accordance with our data, we observed that PDGF-BB decreased SMC α -actin expression. More importantly, our results demonstrated that CBA treatment partly rescued the expression of SMC α-actin (Supplementary Figure S3). These results suggest that CBA halts the change toward a noxious VSMC phenotype induced by PDGF-BB, which in turn contributes to the suppression of neointima formation.

In conclusion, our study demonstrated that CBA inhibits the migration of cultured VSMCs, which may be a major inducer of atherosclerosis and restenosis, *in vitro*. We demonstrate that CBA regulates molecules involved in vessel wall remodeling and/or the accumulation of cells and ECM. Therefore, our findings may provide a novel strategy for inhibiting VSMC migration under pathological conditions, thus offering a novel therapeutic approach for the treatment of restenosis and other vascular diseases involving VSMC migration.

## Materials and Methods

### Cell cultures

HASMCs were purchased from Applied Biological Materials Inc. (Cat. no. T4050) and were maintained in Prigrow III medium (Applied Biological Materials Inc.; Cat. no. TM003) in the presence of 5% fetal bovine serum (FBS) (Cat. no. SH30919.03; HyClone, Logan, UT, USA) and penicillin (100 U/ml)/streptomycin (100 μg/ml) (Invitrogen, Carlsbad, CA, USA). MOVAS-1 murine primary aortic VSMCs were obtained from the American Type Culture Collection (ATCC; Cat. no CRL-2797™) and were cultured in Dulbecco’s modified Eagle’s medium (DMEM; Invitrogen) supplemented with 10% FBS, G418 (0.2 mg/ml) and penicillin (100 U/ml)/streptomycin (100 μg/ml) at 37 °C with a 5% CO2 atmosphere in a humidified incubator.

### Reagents

CBA was obtained from Sigma (St. Louis, MO, USA). The structure of CBA is illustrated in Fig. 1a. The 3-(4,5-Dimethylthiazol‑2‑yl)-2,5-diphenyltetrazolium bromide (MTT) was from Sigma (St. Louis, MO, USA). PDGF‑BB was obtained from PeproTech (Seoul, Korea). Gelatin was obtained from Difco (Lexington, KY, USA). Lipofectamine 2000 reagent was purchased from Invitrogen. Anti-p-PDGFRβ (Cat. no. 4549) and anti-p‑Akt (Ser473; Cat. no. 4058) antibodies were obtained from Cell Signaling Technology, Inc. (Beverly, MA, USA). Anti-p-ERK (Cat. no. sc‑7383), anti-ERK (Cat. no. sc‑93), anti-Akt (Cat. no. sc‑5298) and anti-α-tubulin (Cat. no. sc‑5286) antibodies were from Santa Cruz Biotechnology, Inc. (Santa Cruz, CA, USA). All the chemicals not mentioned above were obtained from Sigma.

### Cell viability

Cell viability was measured by MTT assay. The cells were seeded into 96-well plates at a density of 2 × 10^5^ cells/well. Following serum starvation for 24 h, the cells were treated with PDGF‑BB (10 ng/ml) in the presence of 1–50 μM CBA for 30 min. Following incubation for 24 h at 37 °C, 20 μl of 5 mg/ml MTT solution was added, and the cells were incubated for 1 h at 37 °C, followed by the addition of 100 μl dimethyl sulfoxide (DMSO) to each well to dissolve the formazan. The absorbance was measured at 550 nm using a microplate reader (Magellan; Tecan). Cell viability was expressed as a percentage of the absorbance value determined for the control cultures.

### Western blot analysis

To examine the protein expression patterns, equal amounts (15 μg) of total protein extracts were prepared. The cell lysates were separated on 9% SDS-polyacrylamide gels and transferred onto nitrocellulose membranes. The blots were then incubated with antibodies (anti-p-PDGF-R, anti-p‑ERK, anti-ERK, anti-p-Akt and anti-Akt) and detected using the enhanced chemiluminescence detection system (Amersham Pharmacia Biotech, Piscataway, NJ, USA). The same blots were then stripped and reprobed with anti-tubulin antibody for use as an internal control. Quantitative analysis of the results of western blot analysis were carried out using ImageJ software.

### Wound healing assay

The cells were incubated until they reached 90–100% confluence in 12-well plates. Subsequently, a scratch was gently made using a P-10 pipette tip, and the cells were then treated with 1–10 μM CBA with PDGF‑BB. The cells were subsequently allowed to migrate for 24 h (MOVAS-1) or 48 h (HASMCs) and incubated for various periods of time, subsequently, their phase contrast images were acquired using a Nikon microscopy system (Nikon Instruments Inc., Melville, NY, USA), and measurement of the healed wound gap distance was performed using ImageJ software.

### Reverse transcription-polymerase chain reaction (RT-PCR)

Total RNA was extracted from the cells using TRIzol reagent (Invitrogen) according to the manufacturer’s instructions. Approximately 2 μg of total RNA was used to prepare cDNA using the SuperScript First Strand cDNA Synthesis kit (Bioneer Corporation, Daejeon, South Korea). The following primers were used in this study: mMMP-2 forward, 5′-AAGGATGGACTCCTGGCACATGCCTTT-3′ and reverse, 5′-ACCTGTGGGCTTGTCACGTGGTGT-3′; mGAPDH forward, 5′-GGAGCCAAAAGGGTCATCAT-3′ and reverse, 5′-GTGATGGCATGGACTGTGGT-3′; hMMP-2 forward, 5′-ATGACAGCTGCACCACTGAG-3′ and reverse, 5′-ATTTGTTGCCCAGGAAAGTG-3′; and hGAPDH forward, 5′-CCATCACCATCTTCCAGGAG-3′ and reverse, 5′-CCTGCTTCACCACGTTCTTG-3′. PCR was performed with Platinum Taq polymerase (Invitrogen) under the following conditions: 30 cycles of 96 °C for 40 sec, 55 °C (MMP-2) or 60 °C (GAPDH) for 40 sec, 72 °C for 1 min followed by 10 min at 72 °C. The PCR products were electrophoresed on a 2% (w/v) agarose gel in 1 × Tris‑acetate‑EDTA (TAE) buffer, and stained with ethidium bromide solution. All the PCR reactions were repeated at least three times. GAPDH was amplified as an internal control. The intensity of each band amplified by RT-PCR was analysed using MultiImage™ Light Cabinet (version 5.5; Alpha Innotech Corp., San Leandro, CA, USA), and normalised to that of GAPDH mRNA in corresponding samples.

### Luciferase reporter gene activity assay

Cells were seeded in 12 well plates and transiently transfected with MMP-2 luciferase plasmids using Lipofectamine 2000 reagent (Invitrogen). Cells were then washed twice with ice-cold PBS and lysed in the culture dishes with reporter lysis buffer (Promega Corporation, Madison, WI). The Luciferase assay was performed using the Luciferase Assay System (Promega). Luciferase activity was recorded in a Luminometer 20/20^n^ (Turner BioSystems, Sunnyvale, CA), according to the manufacturer’s instructions. Luciferase activity was normalised with β-galactosidase activity. All data were presented as the mean ± SE of a least three independent experiments.

### Gelatin zymography

The presence of MMP-2 in the supernatants of CBA- and/or PDGF‑BB-treated SMCs was analysed using gelatin zymograms. In briefly, the cells were incubated in serum-free DMEM and the supernatants were collected following incubation for 24 h at 37 °C, clarified by centrifugation (13,000 rpm for 5 min at 4 °C), normalised to the total protein concentration of the cell lysate, mixed with non-reducing Laemmli smaple buffer and separated by electrophoresis on 10% SDS-PAGE gels containing 1 mg/ml gelatin (Difco). Following electrophoresis, the gels were re-natured by washing in 2.5% Triton X-100 solution twice for 30 min to remove SDS. The gels were then incubated in 50 mmol/l Tris-HCl (pH 7.4), 5 mmol/l CaCl2 and 1 μM ZnCl2 at 37 °C overnight. Following incubation, the gels were stained with 0.05% Coomassie brilliant blue R-250 for 30 min at room temperature and then destained in distilled water. MMP-2 activities were visible as clear bands on a blue background where the gelatin substrate had been hydrolysed by enzyme activity.

### ELISA for MMP-2

The supernatants were collected for measuring the amount of secreted MMP-2 protein. The total MMP-2 protein was assayed according to the instructions provided with the Quantkine ELISA kit (R&D Systems, Inc., Minneapolis, MN, USA). In brief, samples and MMP-2 standards were added to microplates pre‑coated with antibody specifically recognising both the pro- and active forms of MMP‑2. After washing, bound MMP‑2 was measured using a horseradish peroxidase‑conjugated secondary anti‑MMP‑2 antibody, developed with H_2_O_2_ and tetramethylbenzidine. The optical density was measured at 450 nm using a Bio‑Rad Model 550 microplate reader and associated Microplate Manager software (Bio‑Rad Laboratories, Mississauga, ON, Canada).

### Aortic ring assay

The *ex vivo* migration and proliferation of VSMCs were measured by aortic ring assay using Matrigel. Male C57Bl/6 J mice (n = 6 in each group), which were 6–8 weeks of age, were purchased from Orient Bio, Inc. (Seoul, Korea) and housed under specific pathogen‑free conditions. All animals were treated in accordance with the Animal Care Guidelines of Use of Laboratory Animals and approved by the Laboratory of Animal Research in Asan institute of Life Sciences. For the *ex vivo* VSMC migration and proliferation assays the mice which were sacrificed by prolonged exposure to isoflurane followed by cervical dislocation, then thoracic aortas were removed and immediately transferred to a culture dish containing serum-free DMEM. The peri‑aortic fibroadipose tissue was carefully removed with fine microdissecting forceps and iridectomy scissors, paying special attention not to damage the aortic wall. One millimetre-thick aortic rings, approximately 12 per aorta, were sectioned and extensively rinsed in five consecutive washes of serum-free DMEM. These rings were embedded in Matrigel (BD Biosciences, Franklin Lakes, NJ, USA) in 48-well plates. Simultaneously, CBA (0–10 μM) and PDGF-BB (10 ng/ml) were added to the culture medium for 3 days. The ring formation images were acquired using a ZEISS microscope (Carl Zeiss, Oberkochen, Germany), and the length of the sprouts was analysed.

All animal procedures were reviewed and approved by the Institutional Animal Care and Use Committee of University of Ulsan College of Medicine (approval no. 2014‑12‑140) and were performed in strict accordance with the Association for Assessment and Accreditation of Laboratory Animal Care and the NIH guidelines (Guide for the Care and Use of Laboratory Animals).

### Rat carotid artery BI model

Male Sprague-Dawley rats (250–300 g; Orient-Charles River Technology, Seoul, Korea) were separated into three groups: a normal control group. A BI group, and a CBA-treated goup (n = 6 for each group). Briefly, under Zoletil (20 mg/kg) and Rompun (5 mg/kg) anesthesia, the left carotid artery was isolated, and a 2-Fr Fogarty ballon catheter (Baxter Healthcare Corp.) was introduced through an external carotid arteriotomy incision, advanced to the aortic arch, inflated to produce moderate resistance, and gradually withdrawn three times. Then, the catherter was removed, and the proximal external carotid artery was ligated. Sham operations were performed on the right common carotid arteries. For the CBA treated group, CBA at a final blood concentration of 2 μM, which was considered plasma concentration in mice blood^25^, was intravenously injected through femoral vein. At 14d after BI, the rats were anesthetized, and the carotid arteries were excised. The entire length of the right carotid artery was balloon injured. The left carotid artery served as an uninjured intra-animal control. To assess the neointima formation, H & E stained section was imaged, and the intima to media thickness ration was measured.

### Statistical analysis

Statistical analysis was performed using the computer program Prism (GraphPad Software, Inc., La Jolla, CA, USA). The results are presented as the means ± SE. Statistical analysis was performed using one-way analysis of variance, followed by Dunnett’s multiple comparison tests. A P-value < 0.05 was considered to indicate a statistically significant difference.

## Electronic supplementary material

Supplementary Dataset

## References

[1] Ross R (1993). The pathogenesis of atherosclerosis: a perspective for the 1990s. Nature.

[2] Hansson GK, Hermansson A (2011). The immune system in atherosclerosis. Nat Immunol.

[3] Libby P, Ridker PM, Hansson GK (2011). Progress and challenges in translating the biology of atherosclerosis. Nature.

[4] Bailey SR (2002). Coronary restenosis: a review of current insights and therapies. Catheter Cardiovasc Interv.

[5] Gerthoffer WT (2007). Mechanisms of vascular smooth muscle cell migration. Circ Res.

[6] Abedi H, Zachary I (1995). Signalling mechanisms in the regulation of vascular cell migration. Cardiovasc Res.

[7] Siefert SA, Sarkar R (2012). Matrix metalloproteinases in vascular physiology and disease. Vascular.

[8] Rubin K (1988). Induction of B-type receptors for platelet-derived growth factor in vascular inflammation: possible implications for development of vascular proliferative lesions. Lancet.

[9] Evanko SP, Raines EW, Ross R, Gold LI, Wight TN (1998). Proteoglycan distribution in lesions of atherosclerosis depends on lesion severity, structural characteristics, and the proximity of platelet-derived growth factor and transforming growth factor-beta. Am J Pathol.

[10] Raines EW (2004). PDGF and cardiovascular disease. Cytokine Growth Factor Rev.

[11] Heldin CH, Westermark B (1999). Mechanism of action and *in vivo* role of platelet-derived growth factor. Physiol Rev.

[12] Zhan Y (2003). Role of JNK, p38, and ERK in platelet-derived growth factor-induced vascular proliferation, migration, and gene expression. Arterioscler Thromb Vasc Biol.

[13] Muto A (2007). Smooth muscle cell signal transduction: implications of vascular biology for vascular surgeons. J Vasc Surg.

[14] Seo J (2011). Tangeretin, a citrus flavonoid, inhibits PGDF-BB-induced proliferation and migration of aortic smooth muscle cells by blocking AKT activation. Eur J Pharmacol.

[15] Lee SJ (2008). 4-Hydroxynonenal enhances MMP-2 production in vascular smooth muscle cells via mitochondrial ROS-mediated activation of the Akt/NF-kappaB signaling pathways. Free Radic Biol Med.

[16] Seo KW (2010). Participation of 5-lipoxygenase-derived LTB(4) in 4-hydroxynonenal-enhanced MMP-2 production in vascular smooth muscle cells. Atherosclerosis.

[17] Southgate KM, Mehta D, Izzat MB, Newby AC, Angelini GD (1999). Increased secretion of basement membrane-degrading metalloproteinases in pig saphenous vein into carotid artery interposition grafts. Arterioscler Thromb Vasc Biol.

[18] Newby AC (2006). Matrix metalloproteinases regulate migration, proliferation, and death of vascular smooth muscle cells by degrading matrix and non-matrix substrates. Cardiovasc Res.

[19] Uzui H, Lee JD, Shimizu H, Tsutani H, Ueda T (2000). The role of protein-tyrosine phosphorylation and gelatinase production in the migration and proliferation of smooth muscle cells. Atherosclerosis.

[20] Johnson C, Galis ZS (2004). Matrix metalloproteinase-2 and -9 differentially regulate smooth muscle cell migration and cell-mediated collagen organization. Arterioscler Thromb Vasc Biol.

[21] Ricci C, Ferri N (2015). Naturally occurring PDGF receptor inhibitors with potential anti-atherosclerotic properties. Vascul Pharmacol.

[22] Yi ZC, Liu YZ, Li HX, Wang Z (2009). Chebulinic acid and tellimagrandin I inhibit DNA strand breaks by hydroquinone/Cu(II) and H(2)O(2)/Cu(II), but potentiate DNA strand breaks by H(2)O(2)/Fe(II). Toxicol In Vitro.

[23] Afshari AR, Sadeghnia HR, Mollazadeh H (2016). A Review on Potential Mechanisms of Terminalia chebula in Alzheimer’s Disease. Adv Pharmacol Sci.

[24] Yi ZC (2004). Effects of chebulinic acid on differentiation of human leukemia K562 cells. Acta Pharmacol Sin.

[25] Lu K (2012). Triphala and its active constituent chebulinic acid are natural inhibitors of vascular endothelial growth factor-a mediated angiogenesis. PLoS One.

[26] Yang MH, Ali Z, Khan IA, Khan SI (2014). Anti-inflammatory activity of constituents isolated from Terminalia chebula. Nat Prod Commun.

[27] Guan YY, Kwan CY, Hsu FL, Cheng JT (1996). *In vitro* inhibitory effects of chebulinic acid on the contractile responses of cardiovascular muscles. Clin Exp Pharmacol Physiol.

[28] Sivasankar S, Lavanya R, Brindha P, Angayarkanni N (2015). Aqueous and alcoholic extracts of Triphala and their active compounds chebulagic acid and chebulinic acid prevented epithelial to mesenchymal transition in retinal pigment epithelial cells, by inhibiting SMAD-3 phosphorylation. PLoS One.

[29] Doran AC, Meller N, McNamara CA (2008). Role of smooth muscle cells in the initiation and early progression of atherosclerosis. Arterioscler Thromb Vasc Biol.

[30] Beaudeux JL, Giral P, Bruckert E, Foglietti MJ, Chapman MJ (2003). [Matrix metalloproteinases and atherosclerosis. Therapeutic aspects]. Ann Biol Clin (Paris).

[31] Kuzuya M (2006). Effect of MMP-2 deficiency on atherosclerotic lesion formation in apoE-deficient mice. Arterioscler Thromb Vasc Biol.

[32] Gharibi B, Ghuman MS, Hughes FJ (2012). Akt- and Erk-mediated regulation of proliferation and differentiation during PDGFRbeta-induced MSC self-renewal. J Cell Mol Med.

[33] Long LH, Hoi A, Halliwell B (2010). Instability of, and generation of hydrogen peroxide by, phenolic compounds in cell culture media. Arch Biochem Biophys.

[34] Clement MV, Ramalingam J, Long LH, Halliwell B (2001). The *in vitro* cytotoxicity of ascorbate depends on the culture medium used to perform the assay and involves hydrogen peroxide. Antioxid Redox Signal.

[35] Lee KW, Hur HJ, Lee HJ, Lee CY (2005). Antiproliferative effects of dietary phenolic substances and hydrogen peroxide. J Agric Food Chem.

[36] Majesky MW (1990). PDGF ligand and receptor gene expression during repair of arterial injury. J Cell Biol.

[37] Kang H (2016). Magnobovatol inhibits smooth muscle cell migration by suppressing PDGF-Rbeta phosphorylation and inhibiting matrix metalloproteinase-2 expression. Int J Mol Med.

[38] Risinger GM, Hunt TS, Updike DL, Bullen EC, Howard EW (2006). Matrix metalloproteinase-2 expression by vascular smooth muscle cells is mediated by both stimulatory and inhibitory signals in response to growth factors. J Biol Chem.

[39] Kuzuya M, Iguchi A (2003). Role of matrix metalloproteinases in vascular remodeling. J Atheroscler Thromb.

[40] Owens GK, Kumar MS, Wamhoff BR (2004). Molecular regulation of vascular smooth muscle cell differentiation in development and disease. Physiol Rev.

[41] Guan H (2012). 3,3’Diindolylmethane suppresses vascular smooth muscle cell phenotypic modulation and inhibits neointima formation after carotid injury. PLoS One.

[42] Rensen SS, Doevendans PA, van Eys GJ (2007). Regulation and characteristics of vascular smooth muscle cell phenotypic diversity. Neth Heart J.

