# Supplementary material for: Chebulinic acid inhibits smooth muscle cell migration by suppressing PDGF-Rβ phosphorylation and inhibiting matrix metalloproteinase-2 expression
Source: Sci Rep. 2017 Sep 18;7:11797. doi: 10.1038/s41598-017-12221-w (PMC5603554; doi:10.1038/s41598-017-12221-w)
Supplement: Supplementary file 1 — Supplementary Dataset [file 41598_2017_12221_MOESM1_ESM.doc]

**Supplementary Information**

**Chebulinic acid** **inhibits smooth muscle cell migration by suppressing PDGF-Rβ phosphorylation and inhibiting matrix metalloproteinase-2 expression**

By

# In-Sung Song^a, b^, Yu Jeong Jeong^a, b^, Jung-Hyun Park^a, b^, Sungbo Shim^c^, *, and Sung-Wuk Jang^a, b^, *

# Supplementary Figures

**Figure S1**

**
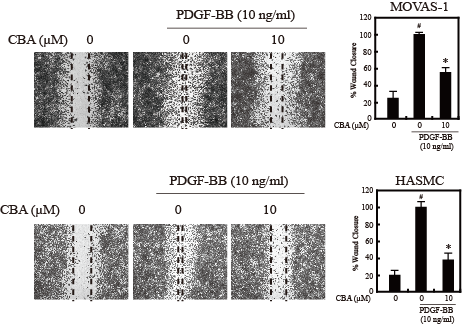
**

**Supplementary Figure S1: CBA inhibits the PDGF-induced VSMC migration:** HASMCs (A) and MOVAS-1 cells (B) were scratched with a pipette tip and were incubated with PDGF-BB (10 ng/ml) in serum-free medium. After 60 min, the cells were treated with CBA for 24 h (MOVAS-1) or 48 h (HASMCs). Representative images of wound healing were taken at the time of scratching and 24 h (or 48 h) after wounding. Original magnification, 100×. Scale bar, 100 μm. Wound healing was quantified as the percentage of cells migrating into the wound with respect to the total number of cells. ***#****P* < 0.001 versus vehicle-treated cells, ********P* < 0.01 versus PDGF-treated cells. Data represent the mean + SE of three independent experiments.

# Figure S2

#
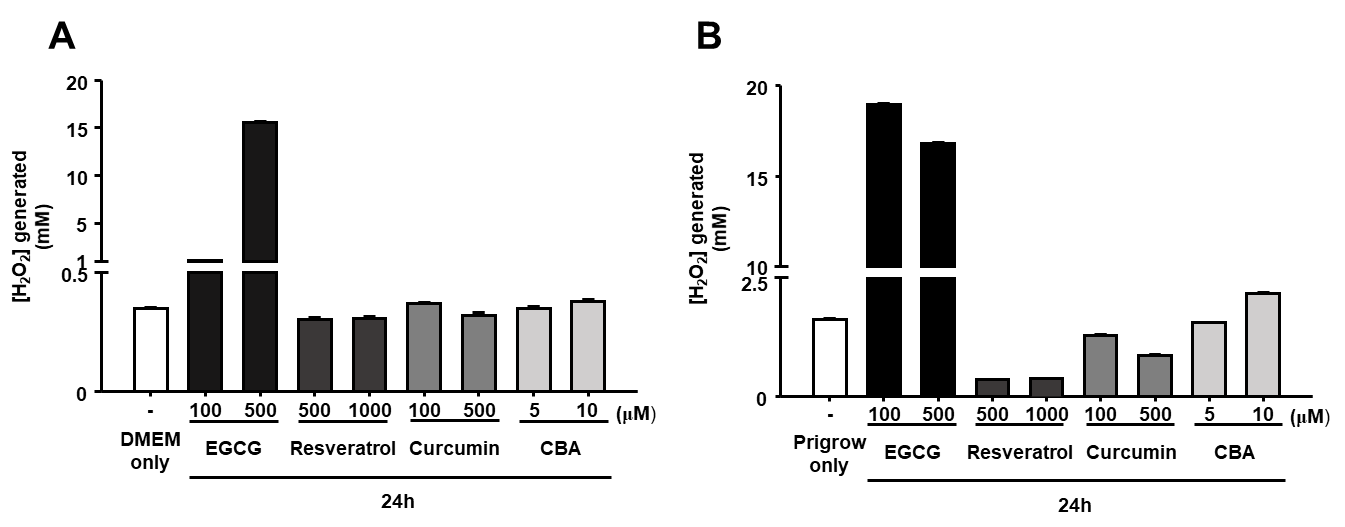


**Supplementary Figure S2: Generation of H_2_O_2_ in culture medium by EGCG, resveratrol, curcumin, and CBA:** A) Generation of H_2_O_2_ by EGCG, resveratrol, curcumin, and CBA in DMEM including 10% FBS for 24 h. H_2_O_2_ was measured by the Amplex® Red Hydrogen Peroxide/Peroxidase Assay Kit. B) Generation of H_2_O_2_ by EGCG, resveratrol, curcumin, and CBA in Prigrow media including 5% FBS for 24 h. H_2_O_2_ was measured by the Amplex® Red Hydrogen Peroxide/Peroxidase Assay Kit.

**Figure S3**

**
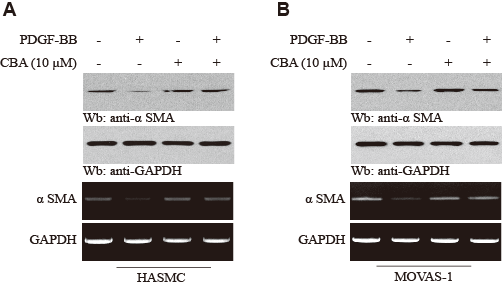
**

**Supplementary Figure S2: CBA inhibits the PDGF-induced VSMC synthetic phenotype:** HASMC (A) and MOVAS-1 (B) pre-incubated for 30 min with CBA and then stimulated with PDGF-BB (10 ng/ml) for 24 h. The mRNA and protein level of α-SMA was determined with RT-PCR and western blot analysis.

**Supplementary Materials and Methods**

**Antibody**

Anti-smooth muscle α-actin antibody (ab5694) was obtained from Abcam (Cambridge, MA)

**Measurement of the concentration of H_2_O_2_ in serum-containing culture media.**

EGCG, resveratrol, curcumin, and CBA were added to 6-well culture plates filled with 2 ml of DMEM or Prigrow, and the plates were placed at 37˚C under 5% CO_2_. After 24h, part of the medium was collected to measure the concentration of H_2_O_2_ by the Amplex Red hydrogen peroxide/peroxidase assay kit (Invitrogen). Briefly, medium (50 μl) was mixed with 50 μl of reaction mixture (50 mM Amplex Red reagent, 0.1 U horsearadish peroxidase ml-1 in 0.5 M sodium phosphate buffer, pH 7.4) was dispensed into wells of a 96-well microtitre plate and warmed to 37˚C for 20 min. After 20 minutes, the absorbance was measured at 560 nm (Powerwave XS Microplate Spectrophotometer, Bio Tek Instruments, Winooski, USA) according to the manufacturer’s instructions in order to determine the peroxidase activity of EGCG, resveratrol, curcumin, and CBA, respectively.
